# Evolution of Sex-Specific Traits through Changes in HOX-Dependent *doublesex* Expression

**DOI:** 10.1371/journal.pbio.1001131

**Published:** 2011-08-23

**Authors:** Kohtaro Tanaka, Olga Barmina, Laura E. Sanders, Michelle N. Arbeitman, Artyom Kopp

**Affiliations:** 1 Department of Evolution and Ecology, University of California–Davis, Davis, California, United States of America; 2 Section of Molecular and Computational Biology, Department of Biological Sciences, University of Southern California, Los Angeles, California, United States of America; Stowers Institute for Medical Research, United States of America

## Abstract

Almost every animal lineage is characterized by unique sex-specific traits, implying that such traits are gained and lost frequently in evolution. However, the genetic mechanisms responsible for these changes are not understood. In *Drosophila*, the activity of the sex determination pathway is restricted to sexually dimorphic tissues, suggesting that spatial regulation of this pathway may contribute to the evolution of sex-specific traits. We examine the regulation and function of *doublesex* (*dsx*), the main transcriptional effector of the sex determination pathway, in the development and evolution of *Drosophila* sex combs. Sex combs are a recent evolutionary innovation and show dramatic diversity in the relatively few *Drosophila* species that have them. We show that *dsx* expression in the presumptive sex comb region is activated by the HOX gene *Sex combs reduced* (*Scr*), and that the male isoform of *dsx* up-regulates *Scr* so that both genes become expressed at high levels in this region in males but not in females. Precise spatial regulation of *dsx* is essential for defining sex comb position and morphology. Comparative analysis of *Scr* and *dsx* expression reveals a tight correlation between sex comb morphology and the expression patterns of both genes. In species that primitively lack sex combs, no *dsx* expression is observed in the homologous region, suggesting that the origin and diversification of this structure were linked to the gain of a new *dsx* expression domain. Two other, distantly related fly lineages that independently evolved novel male-specific structures show evolutionary gains of *dsx* expression in the corresponding tissues, where *dsx* may also be controlled by *Scr*. These findings suggest that changes in the spatial regulation of sex-determining genes are a key mechanism that enables the evolution of new sex-specific traits, contributing to some of the most dramatic examples of phenotypic diversification in nature.

## Abstract 2

Analysis in *Drosophila* suggests that evolutionary changes in the spatial regulation of the transcription factor doublesex play a key role in the origin, diversification, and loss of sex-specific structures.

## Author Summary

Most animals are sexually dimorphic, yet each species has a different set of sex-specific traits. Much of evolutionary biology since Darwin has focused on explaining these differences. In contrast to the well-developed theories of sexual selection (how and why males compete for females) we are still far from understanding the molecular mechanisms underlying the rapid gain and loss of sexually dimorphic phenotypes. In *Drosophila melanogaster*, the development of most sex-specific traits is controlled by the *doublesex* transcription factor. One of these traits is the sex comb, a group of modified bristles that develops on the front legs of males, which they use during mating to grasp the female's abdomen and genitalia. Sex combs are a recent innovation that evolved within the genus *Drosophila* but show dramatic diversity in the relatively few species that have them. In this study, we show that the origin and diversification of sex combs were associated with an evolutionary gain of a new *doublesex* expression domain and novel regulatory interactions between *doublesex* and the HOX gene *Sex combs reduced*, best known for its role in the specification of the labial and first thoracic segments. We find that other sex-specific structures that evolved in separate *Drosophila* lineages are also linked to new *doublesex* expression domains, suggesting that changes in the spatial regulation of *doublesex* may be a general mechanism enabling the evolutionary turnover of sex-specific traits.

## Introduction

Sexual dimorphism is a common feature of animal morphology. Most lineages display unique sets of sex-specific traits, indicating that new sexual characters are gained and old ones are lost, frequently in evolution. At the genetic level, the origin of sex-specific structures from sexually monomorphic precursors implies the evolution of new, sexually dimorphic regulatory pathways. One way in which this could occur is through the emergence of novel interactions between the sex determination pathway and an ancestrally monomorphic genetic network that controls pattern formation and morphogenesis in the evolving tissue. The nature and origin of such interactions can best be understood by characterizing the development of recently evolved sex-specific traits that have sexually monomorphic homologs in closely related species [1]–[5].

One such trait is the *Drosophila* sex comb, a male-specific structure that develops on the first pair of legs (T1) from stereotypically arranged mechanosensory bristles. The sex comb is a recent evolutionary innovation, present in a relatively small subset of *Drosophila* species including the *melanogaster* and *obscura* species groups [6]–[8]. Following their origin, sex combs have undergone dramatic morphological diversification with many examples of rapid divergence between closely related species and convergent evolution in distantly related ones [9],[10]. This pattern may be caused by sexual selection, since sex combs are used by males for grasping and stimulating females during mating [11]–[14]. Sex combs can develop by different cellular mechanisms, including a coordinated rotation of the surrounding epithelium [15]–[18]. The presence of sex combs in the model species *D. melanogaster*, their diversity among close relatives of this species, and the existence of more distant *Drosophila* lineages that primitively lack sex combs make this structure an excellent model for investigating the developmental mechanisms responsible for the origin and diversification of novel sex-specific traits.

In *Drosophila*, sexual differentiation of most somatic tissues is controlled by the sex-specific transcription factors encoded by *doublesex* (*dsx*), an effector of the sex determination pathway that is regulated by alternative pre-mRNA splicing [19]–[21]. The male isoform (*dsxM*) promotes the development of male-specific structures, including the sex comb, and represses female-specific structures; the female isoform (*dsxF*) promotes female-specific and represses male-specific traits [22]–[25]. DsxM and DsxF proteins share a common N-terminal DNA-binding domain, but have different C-terminal domains that have different effects on target gene expression [2],[26]–[29].

Recent studies have shown that *dsx* is not only regulated at the level of sex-specific splicing, but is also expressed in precisely defined spatial patterns in the gonad, CNS, and other tissues [30]–[35]. These observations suggest that sexual identity is only interpreted by the subset of cells that undergo sex-specific differentiation [35],[36]. However, the regulatory mechanisms that control the spatial pattern of *dsx* expression, and the importance of spatial regulation of *dsx* in the development and evolution of sexually dimorphic structures, are not understood.

In this report, we show that precise spatial regulation of *dsx* is essential for sex comb development and has played a key role in the origin and evolution of this structure. The sex comb of *D. melanogaster* develops from a transverse bristle row that is present in both sexes but undergoes male-specific morphogenesis including a 90 degree rotation and a strong modification of the individual bristle shafts (“teeth”) [15],[16],[18]. Sex comb development requires both *dsx* and the HOX gene *Sex combs reduced* (*Scr*), suggesting that *dsx* and *Scr* cooperate to induce sex- and segment-specific downstream targets [24],[37]. We now show that *Scr* acts in part by activating localized *dsx* expression in the presumptive sex comb region. *dsx* and *Scr* then establish an autoregulatory loop that drives sexually dimorphic differentiation of the sex comb and surrounding epidermal cells. In *Drosophila* species that primitively lack sex combs, *dsx* is not expressed in the homologous region of the T1 leg, while in species that do have sex combs the spatial patterns of *dsx* and *Scr* reflect sex comb position and morphology. Our results suggest that the origin of a new *dsx* expression domain, and the evolution of the *dsx*-*Scr* feedback loop, have led to the emergence and diversification of this novel sex-specific structure. We propose that similar mechanisms based on the spatial regulation of sex-determining genes may contribute to the origin of new sex-specific traits in other animals. Consistent with this hypothesis, *dsx* shows specific, derived expression patterns in two other Drosophilid lineages that independently evolved different male-specific structures on their legs.

## Results

### *dsx* Expression Is Regulated in a Spatio-Temporal and Sex-Specific Manner

**Figure 1:**
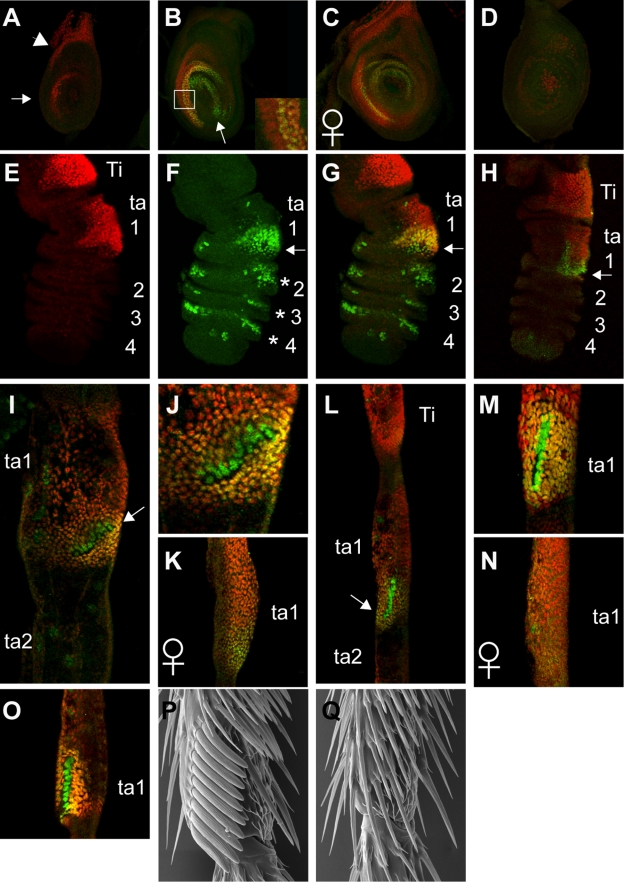
Dsx and Scr expression during sex comb development in *D. melanogaster*. Immunostaining with anti-Scr (red) and anti-DsxC (green) antibodies. Ti, tibia; ta, tarsus; AP, after pupariation. All panels except (E) and (F) are merged images. (A) L3 male T1 leg disc. Anterior is to the left, dorsal is up. Scr is expressed at a high level in the anterior part of distal Ti and ta1 (arrow) and in a more proximal region corresponding to the presumptive body wall (arrowhead). Low expression is present in the rest of the disc. (B) Wandering male T1 leg disc. Dsx is expressed in the distal part of the Scr domain (overlap in yellow) and in the more central region (arrow). Inset shows a magnified view of the boxed area. (C) Wandering female T1 leg disc. (D) Wandering male T2 leg disc. The only detectable Scr expression is subepidermal. (E–H) 5 h AP T1 leg (E–G, male; H, female). The tarsal segments are numbered. Dsx is strongly expressed on the ventral-anterior side of distal ta1 in both sexes (arrow). (F) In the male, Dsx is expressed in the distal ta1 and in small dorsal and ventral patches in ta2–4 (asterisks). (H) In the female, Dsx expression is only in the distal ta1 and is weaker than in the male. (I–K) 16 h AP T1 leg (I, J, male; K, female). Arrows point to the rotating sex comb. Note the absence of Dsx expression in ta2. High background staining is caused by the pupal cuticle, which is still attached to the epidermis at this stage. (L–N) 24 h AP T1 leg. (L, M, male; N, female). (O) 36 h AP male T1 leg. (P, Q) Scanning electron micrographs of the distal ta1 in the adult male (P) and female (Q). Ventral is to the right and anterior is facing out of the page.

In *D. melanogaster*, the anterior-ventral side of the first tarsal segment (ta1) is covered with tightly packed transverse bristle rows (TBRs) in both sexes. In the male, the most distal TBR is modified into the sex comb that rotates 90 degrees to align along the proximo-distal leg axis (Figure 1P,Q) [15],[16],[18],[38]. In T1 leg imaginal discs of third instar larvae (L3), *Scr* is strongly expressed in the anterior-ventral region of the presumptive distal tibia (Ti) and ta1 corresponding to the future location of TBRs and the sex comb, and at a lower level in the rest of the disc [10],[39],[40]. *dsx* is expressed in an apparently more restricted domain in the T1 imaginal disc, as indicated by a *dsx*-Gal4 reporter [35]. To characterize the expression pattern of *dsx* during sex comb development in greater detail, we co-stained T1 legs at different stages with antibodies against Scr [39] and the common domain of Dsx (DsxC), which is shared by the male- and female-specific protein isoforms [33].

In late non-wandering L3 T1 leg discs, high levels of Scr are already detectable in the Ti and ta1 region (Figure 1A). In contrast, no Dsx expression is observed at this time in the leg discs of either sex (Figure 1A). By the wandering L3 and white prepupal stages, Dsx expression is apparent in both male and female T1 discs in an anterior-ventral crescent that overlaps the distal but not the proximal part of the high Scr expression domain (Figure 1B,C). In some males, Dsx expression also extends more distally and posteriorly into the region of low Scr expression (Figure 1B); this variability may reflect subtle temporal differences. Dsx expression was not detected in T2 or T3 leg discs (Figure 1D and unpublished data). In the prepupal legs at 5 h after pupariation (5 h AP), Dsx expression is clearly seen in the distal ta1 in both male and female T1 legs (Figure 1E–H). However, the overlap with high Scr expression, which extends more proximally, is more extensive in males than in females (Figure 1G,H). In males, but not in females, Dsx expression is also seen in clusters of cells in the more distal tarsal segments (ta2–ta5) (Figure 1F,G). Thus, Dsx expression becomes sexually dimorphic at the prepupal stage, before the future sex comb bristles are determined.

At 16 h AP, when the sex comb begins its rotation, DsxC expression in the distal ta1 is obviously dimorphic. In males, Dsx is expressed strongly in and around the presumptive sex comb, while female expression is consistently lower (Figure 1I–K). Male-specific expression of Dsx in ta2–ta5 disappears by this time (Figure 1I and unpublished data). By 24 h AP, when sex comb rotation is complete, Dsx and Scr develop roughly complementary expression patterns in the male leg (Figure 1L,M). Dsx is expressed at a high level in sex comb teeth and surrounding epidermal cells, whereas Scr expression is low or absent in sex comb teeth but highest in the adjacent epidermal cells (Figure 1L,M). This pattern is maintained at later stages (Figure 1O). In females, Dsx expression becomes very low or undetectable, and Scr expression in the distal ta1 is much weaker than in males, by 24 h AP (Figure 1N). These observations show that both Dsx and Scr are expressed in tightly restricted and sex-specific patterns in the sex comb at the critical time in its development.

### *dsx* Regulation Is Transcriptional

**Figure 2:**
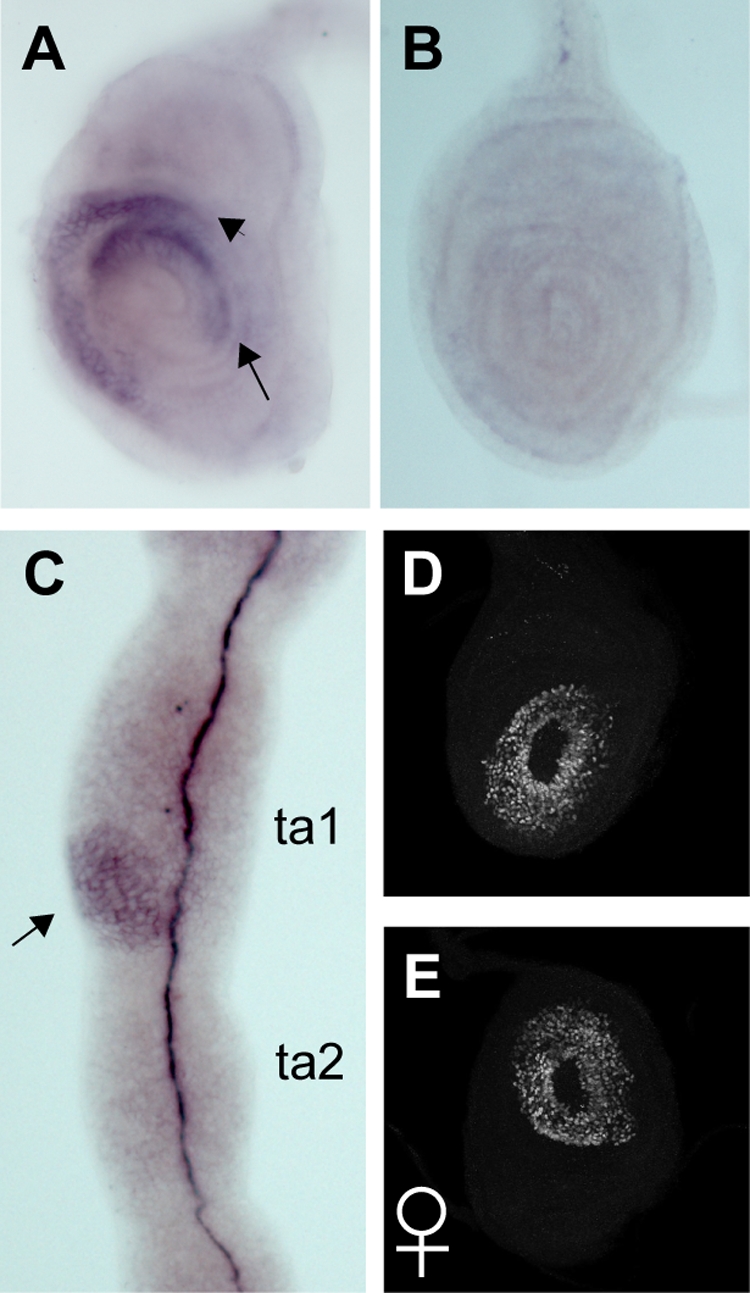
Transcriptional regulation of *dsx*. (A–C) Localization of *dsx* transcripts by in situ hybridization. Anterior is to the left, dorsal is up. (A) In the wandering L3 male T1 leg disc, *dsxM* is expressed in an anterior proximal (arrowhead) and a distal (arrow) crescent domain. (B) No detectable signal is seen in male T2. (C) *dsxM* expression in the 24 h AP male T1 leg. The only epidermal expression is in the presumptive sex comb region (arrow). Strong staining in the center of the leg is non-specific. (D, E) DsxM immunostaining in *rn-Gal4/UAS-dsxM* male (D) and female (E) T1 leg discs is seen throughout the *rn*-expression domain.

The similarities between Dsx protein (Figure 1B,C) and *dsx*-Gal4 [35] expression patterns suggest that the spatially restricted expression of *dsx* in the T1 leg is due to transcriptional regulation. To confirm this, we used an RNA probe directed against the male-specific *dsx* exon to examine *dsxM* expression by in situ hybridization. In wandering L3 and white prepupal leg discs, *dsxM* transcript is present in the same pattern as the Dsx protein (Figure 2A, Figure 1B). This transcript is undetectable either in the male T2 and T3 discs or in the female T1 (Figure 2B). At 24 h AP, *dsxM* transcript in the male T1 leg is confined to the presumptive sex comb region (Figure 2C), similar to the protein distribution (Figure 1M). To confirm that the restriction of *dsxM* to the sex comb region is not due to post-transcriptional regulation, we drove ectopic *UAS-dsxM* expression in both males and females using the *rn-Gal4* driver, which is expressed around the entire circumference of the pupal leg from distal ta1 to ta4 [10]. The *UAS-dsxM* construct [30] contains most of the male-specific 3′UTR, including a predicted recognition site for the *bantam* miRNA. Both in situ hybridization with a *dsxM*-specific probe and immunostaining with the antibody specific to DsxM [31] revealed ectopic *dsxM* expression throughout the tarsus in all three legs, with no detectable difference between males and females (Figure 2D,E). Thus, we find no evidence for a post-transcriptional mechanism confining *dsx* expression to the sex comb region. Finally, quantitative rt-PCR with primers flanking male-specific and female-specific exon junctions did not reveal any differences in *dsx* splicing between T1 and T2 legs (unpublished data). We conclude that the spatially restricted expression of *dsx* in the sex comb region is caused by its precise transcriptional regulation.

### The Roles of *dsx* and *Scr* in Sex Comb Development

**Figure 3:**
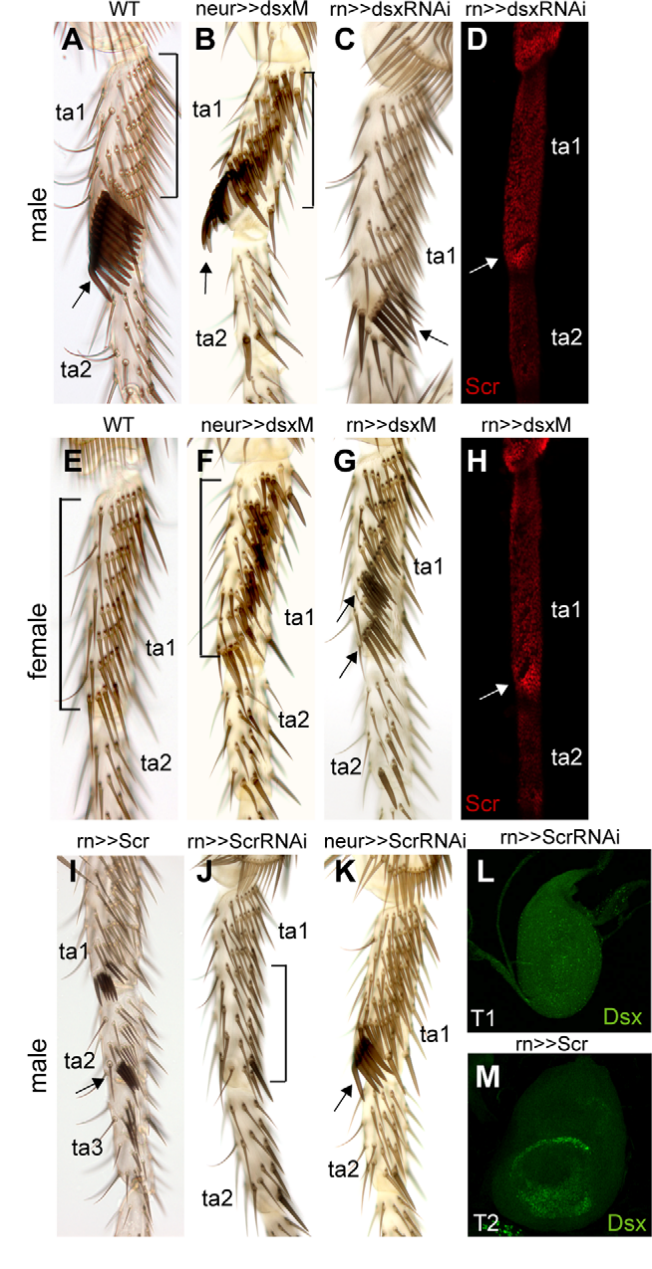
*dsx* and *Scr* control sex comb development. (A) Wild-type male adult T1 leg. ta, tarsus; bracket, TBRs; arrow, sex combs. (B) *tub-Gal80^ts^*; *neur-Gal4/UAS-dsxM* male. The bristles in TBRs are transformed into ectopic sex comb teeth (bracket). Arrow points to the normal sex comb. (C) *tub-Gal80^ts^*; *rn-Gal4/UAS-dsxRNAi* male. The sex comb is only partially rotated and has fewer and thinner teeth (arrow). (D) Scr expression in *tub-Gal80^ts^*; *rn-Gal4/UAS-dsxRNAi* male at 24 h AP. Scr is down-regulated except in the cells distal to the sex comb (arrow). (E) Wild-type female adult T1 leg. Bracket, TBRs. (F) *tub-Gal80^ts^*; *neur-Gal4/UAS-dsxM* female. As in the male of the same genotype (B), TBR bristles assume sex comb-like morphology (bracket). (G) *tub-Gal80^ts^*; *rn-Gal4/UAS-dsxM* female. The two most distal TBRs develop into partially rotated sex combs (arrows). (H) Scr expression in *tub-Gal80^ts^*; *rn-Gal4/UAS-dsxM* female T1 leg at 24 h AP. Scr is up-regulated in cells distal to the ectopic sex comb (arrow); compare to (D). (I) *tub-Gal80^ts^*; *rn-Gal4/UAS-Scr* male. Ectopic sex combs are formed on distal tarsal segments (arrow). (J) *tub-Gal80^ts^*; *rn-Gal4/UAS-ScrRNAi* male. The sex comb and TBR are lost from the distal part of ta1, where *rn* is expressed (bracket). (K) *tub-Gal80^ts^*; *neur-Gal4/UAS-ScrRNAi* male. The number of teeth is reduced, but tooth morphology is normal (arrow). (L) T1 leg disc of *tub-Gal80^ts^/UAS-Gal4*; *rn-Gal4/UAS-ScrRNAi* male. No Dsx is detectable. (M) T2 leg disc of *tub-Gal80^ts^*; *rn-Gal4/UAS-Scr* male at the wandering stage. Ectopic Dsx expression is detected throughout the *rn* expression domain.

To determine the significance of the spatial regulation of *dsx* in sex comb development, we examined the effects of loss and ectopic expression of the male-specific *dsx* isoform (*dsxM*) in different cell types. Knocking down *dsx* in both bristle precursors and epidermal cells in *rn-Gal4/UAS-dsxRNAi* males resulted in an intersex phenotype with small, partially rotated sex combs composed of bristles that were intermediate in morphology between normal sex comb teeth and female TBR bristles (Figure 3C). A similar intersex phenotype was observed in females (not shown). In both sexes, the number of bristles in the partially formed sex comb was intermediate between a wild-type sex comb and the distal-most female TBR. These phenotypes are very similar to the *dsx* null phenotype, confirming that *dsxM* promotes sex comb development in males while *dsxF* actively blocks it in females [22],[35].

Expression of *dsxM* in all leg bristles in *tub-Gal80^ts^*; *neur-Gal4/UAS-dsxM* flies that were shifted to the restrictive temperature as late L3 larvae resulted in the transformation of all or most TBR bristles into sex comb teeth, as indicated by thicker and blunter shafts and dark pigmentation (Figure 3B,F). This phenotype was observed in both males and females. However, none of the bristles outside of the TBRs showed any signs of transformation. In males, the transformation was strongest toward the distal end of ta1, while in females this region showed the weakest transformation (Figure 3B,F). These results suggest that DsxM expression in bristle precursor cells is sufficient to induce sex comb tooth development, but only in regions that express high levels of Scr. The number of bristles in the distal-most female TBR was unchanged despite the changes in bristle morphology. Interestingly, the ventral-posterior region of ta1 in the T3 leg, which also carries TBRs, also developed bristles with sex comb-like morphology; in contrast, no changes were observed in T2 legs (not shown). The T3 TBRs are specified by the HOX gene *Ultrabithorax* [40], while epidermal cells in the T2 leg do not express any HOX genes at the late larval and pupal stages. Thus, it appears that *Ubx* or its downstream targets can substitute for *Scr* in cooperating with *dsxM* to induce sex comb tooth development. In contrast to our results, ectopic expression of *dsxM* under the control of a heat shock promoter (*hs:dsx^M^*) can induce tooth-like bristles in all three legs and in regions outside of the high *Scr* domain in T1 [24]. This difference may be explained by the fact that the *hs:dsx^M^* constructs were expressed throughout development in both bristle and epithelial cells.

Despite the changes in bristle shaft morphology, the proximal TBRs showed little or no rotation in *tub-Gal80^ts^*; *neur-Gal4/UAS-dsxM* flies. We next drove ectopic *dsxM* expression in both the bristles and the epidermal cells in the distal ta1–ta4 in *tub-Gal80^ts^*; *rn-Gal4/UAS-dsxM* flies. In the female, this treatment transformed two to four distal TBRs into small sex combs that underwent complete or partial rotation (Figure 3G). However, the number of bristles per TBR was unchanged. A similar phenotype was observed in males (not shown). No effects were observed in the more distal tarsal segments (Figure 3G). These results confirm that sex comb rotation is driven by the surrounding epidermal cells [16],[18] and that these cells require high levels of both Dsx and Scr. In summary, ectopic expression experiments indicate that *dsxM* acts in bristle precursor cells to specify sex comb tooth morphology and in the surrounding epidermal cells to promote sex comb rotation, and that precise spatial regulation of *dsx* is essential for determining the location and size of this structure.

Next, we investigated cell type-specific requirements for *Scr* in sex comb development. As previously reported [10], uniform *Scr* expression in the distal tarsus in *tub-Gal80^ts^*; *rn-Gal4/UAS-Scr* flies results in the formation of ectopic, non-rotating sex combs in ta2–ta4 in the male T1 leg (Figure 3I). Knocking down *Scr* in *tub-Gal80^ts^*; *rn-Gal4/UAS-ScrRNAi* flies results in the complete loss of the sex comb and TBRs in the distal ta1, indicating a homeotic transformation to the T2 identity (Figure 3J). However, when *Scr* function was knocked down specifically in bristle precursor cells in *tub-Gal80^ts^*; *neur-Gal4/UAS-ScrRNAi* flies, the number of sex comb teeth was reduced to ∼50% of normal, but tooth morphology and rotation were not affected (Figure 3K). This phenotype was not significantly enhanced by the addition of *UAS-Gal4* (not shown). Since Scr expression in ta1 is sexually dimorphic and the sex comb contains more bristles than the homologous female TBR, it is possible that Scr levels determine the number of bristle precursors during larval or prepupal stages. *Scr* may also be required in epidermal cells for sex comb rotation, but is dispensable for the male-specific differentiation of sex comb teeth, at later stages. This is consistent with the observation that Scr protein disappears from the sex comb precursor bristles by 16 h AP (Figure 1LI,J). Thus, many functions of *Scr* in sex comb development may be mediated by the activation of *dsx* expression (see below).

### *Scr* Activates *dsx* Expression in T1

Based on the observations that Dsx is expressed only in the T1 leg disc overlapping the high Scr domain and that Scr expression precedes that of Dsx (Figure 1A–C), we hypothesized that *Scr* positively regulates *dsx* expression. To test this hypothesis, we first performed an RNAi knockdown of *Scr* in *tub-Gal80^ts^*; *rn-Gal4/UAS-ScrRNAi* and *tub-Gal80^ts^/UAS-Gal4*; *rn-Gal4/UAS-ScrRNAi* flies. In both male and female T1 leg discs, DsxC expression was strongly reduced in the former genotype and undetectable in the latter after a 24-h shift to the restrictive temperature (Figure 3L), indicating that *Scr* is necessary for Dsx expression. In a reciprocal experiment, we expressed *Scr* around the entire circumference of distal ta1–ta4 in all three legs in *tub-Gal80^ts^*; *rn-Gal4/UAS-Scr* flies. This resulted in the ectopic expression of Dsx in the same pattern as the ectopic Scr in all three pairs of leg discs (Figure 3M and unpublished data), indicating that *Scr* is sufficient to activate Dsx in the tarsus. These observations suggest that a major role of *Scr* in sex comb development is to initiate a sex-specific developmental program by turning on *dsx* expression. Consistent with this notion, co-expression of *Scr* and *dsxM* in *tub-Gal80^ts^*; *rn-Gal4/UAS-Scr UAS-dsxM* flies produces the same phenotype as ectopic expression of *Scr* alone (not shown).

### *dsx* Modulates *Scr* Expression in the Sex Comb Region

*Scr* expression in the T1 leg is sexually dimorphic in *D. melanogaster* and other species with rotated sex combs (Figure 1L–P) [10]. To test whether *dsx* is responsible for the sex-specific regulation of *Scr*, we first examined the effects of *dsx* knockdown in *rn-Gal4/UAS-dsxRNAi* males. At 24 h AP, Scr expression in the distal ta1 was reduced, becoming intermediate between wild-type male and wild-type female (Figure 3D, compare to Figure 1L–N). In a reciprocal experiment, we looked at the effect of *dsxM* expression in *rn-Gal4/UAS-dsxM* females. In this genotype, Scr expression was induced in the distal ta1 in a pattern identical to the *rn-Gal4/UAS-dsxRNAi* males (Figure 3H). These results are consistent with the effects of *dsx* on adult morphology: in the absence of either *dsxM* or *dsxF*, or in the presence of both isoforms, the distal-most TBR assumes a morphology intermediate between a sex comb and a female TBR in both XX and XY flies (Figure 3C) [22]. We conclude that in the absence of *dsx*, *Scr* is expressed at an intermediate level and that this level is sufficient to induce partial sex comb development in *D. melanogaster*. DsxM up-regulates and DsxF down-regulates *Scr* relative to this default level, so that both isoforms are actively involved in sexually dimorphic development.

### Correlated Evolutionary Changes in *Scr* and *dsx* Expression Are Associated with Sex Comb Diversification

**Figure 4:**
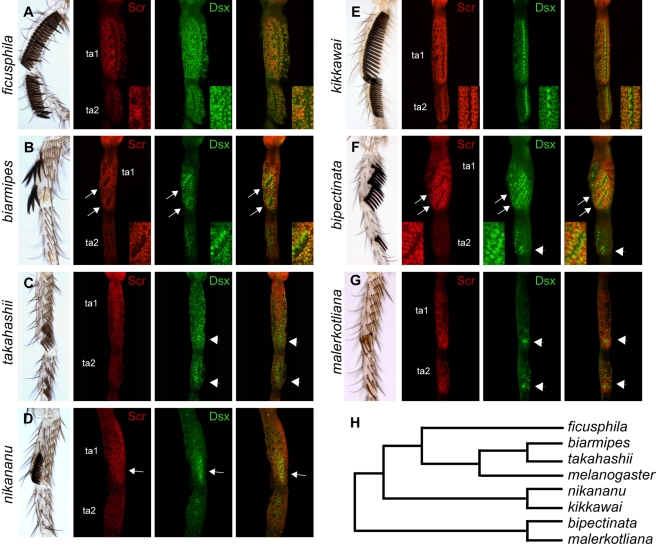
Dsx and Scr expression in the *melanogaster* species group. ta1–2 of adult male T1 legs are shown on the left. Scr (red) and Dsx (green) immunostaining of the same segments in mid-pupal male T1 legs are shown in the right panels. Developing sex combs are indicated by arrows (longitudinal combs) or arrowheads (small and transverse combs). In all species, Dsx expression is highest in the sex comb teeth, while Scr is low in the sex comb teeth but high in the surrounding cells. (A) *D. ficusphila*. (B) *D. biarmipes*. (C) *D. takahashii*. (D) *D. nikananu*. (E) *D. kikkawai*. (F) *D. bipectinata*. (G) *D. malerkotliana*. (H) Phylogenetic relationships among the species shown in this figure. The latest common ancestor of *D. kikkawai* and *D. nikananu* had a sex comb similar to that of *D. kikkawai*; the latest common ancestor of *D. malerkotliana* and *D. bipectinata* had a sex comb similar to *D. malerkotliana* (Barmina and Kopp 2007) [10].

The size and location of sex combs in the *melanogaster* and *obscura* species groups correlate with the domain of high Scr expression [10],[41]. Moreover, Scr expression is sexually dimorphic in species with rotated sex combs, but not in species in which sex comb teeth remain organized into TBRs [10]. We used DsxC and DsxM antibodies to examine *dsx* expression in the presumptive sex comb region in *melanogaster* group species with diverse sex comb morphologies (Figure 4). Importantly, these species represent several independent phylogenetic contrasts, since distantly related species have evolved similar sex combs independently (Figure 4H) [10]. In all species, Dsx expression is strongest in sex comb teeth and is also present in the adjacent epithelial cells, while Scr expression is low or absent in sex comb teeth but highest in the surrounding cells (Figure 4). In *D. ficusphila* and *D. kikkawai*, which independently evolved large sex combs spanning the entire ta1 and ta2, Scr and Dsx are expressed throughout the anterior-ventral surface of these segments (Figure 4A,E). In *D. bipectinata* and *D. biarmipes*, which independently evolved rotated sex combs derived from two separate TBRs, Dsx and Scr are expressed in and around both rows of teeth (Figure 4B,F). In the closest relatives of these species that have transverse sex combs (*D. malerkotliana* and *D. takahashii*, respectively), Dsx expression in epithelial cells is lower than in the species with rotated sex combs, and is only seen in a few cells immediately adjacent to the sex comb (Figure 4C,G). In *D. nikananu*, whose sex comb is secondarily reduced from a *D. kikkawai*-like ancestral state, Dsx expression is also confined to a smaller domain that resembles the *D. melanogaster* pattern (Figure 4D). Thus, the spatial correlation of Dsx and Scr expression is maintained in all species and reflects sex comb morphology rather than phylogenetic history. This pervasive pattern of convergent evolution suggests that the cross-regulatory relationship between Dsx and Scr is conserved throughout the *melanogaster* species group and may contribute to the rapid evolution of sex comb morphology.

### *dsx* Expression in the Presumptive Sex Comb Region Is an Evolutionary Innovation

**Figure 5:**
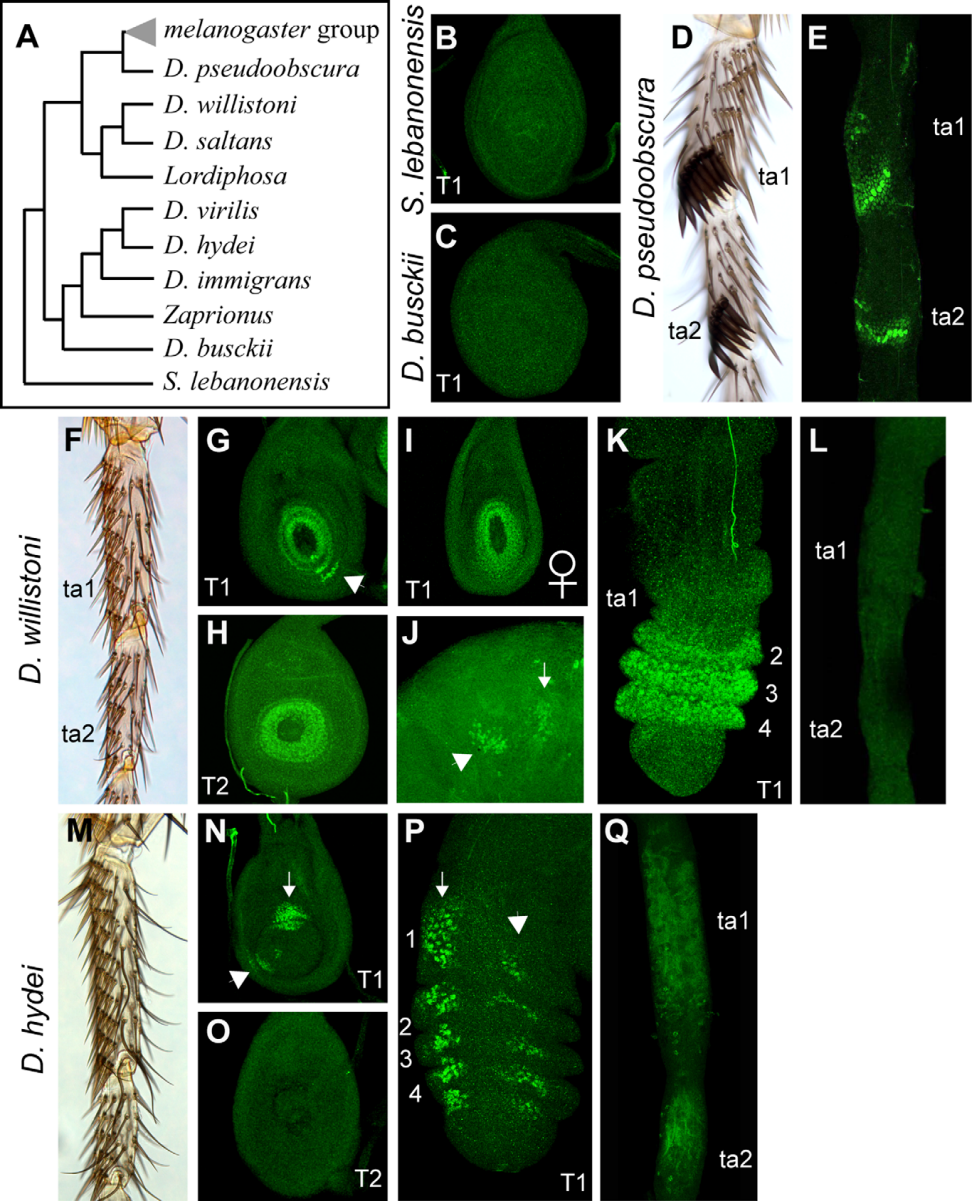
Dsx expression in distantly related lineages. Dsx immunostaining is in green. (A) Simplified phylogeny of the species shown in this figure and Figure 6. (B) Male T1 leg disc of *S. lebanonensis*. (C) Male T1 leg disc of *D. busckii*. (D) Adult male T1 leg of *D. pseudoobscura* carries sex combs on the ta1 and ta2 segments. (E) Dsx expression in the corresponding segments of the male T1 leg at the early pupal stage. (F–L) *D. willistoni*. (F) Adult male T1 leg. Note the absence of sex combs and the very small number of long and curved chemosensory bristles (compare to M). (G) Male T1 leg disc stained with the DsxC antibody. Arrowhead, an expression domain unique to the male T1 disc. (H) Male T2 leg disc stained with the DsxC antibody. (I) Female T1 leg disc stained with the DsxC antibody. (J) Adult male brain stained with the DsxC antibody, showing the PC1 (arrow) and PC2 (arrowhead) neuronal clusters. (K) Male T1 prepupal leg at 5 h AP stained with the DsxC antibody. (L) Male T1 pupal leg at 24 h AP. (M–Q) *D. hydei*. (M) Adult male T1 leg. (N) Male T1 leg disc. Arrow and arrowhead point to the dorsal and ventral expression domains respectively. (O) Male T2 leg disc. (**P**) Male T1 prepupal leg at 8 h AP. The two domains are still visible (arrow, arrowhead). (Q) Male T1 pupal leg at 40 h AP.

**Figure 6:**
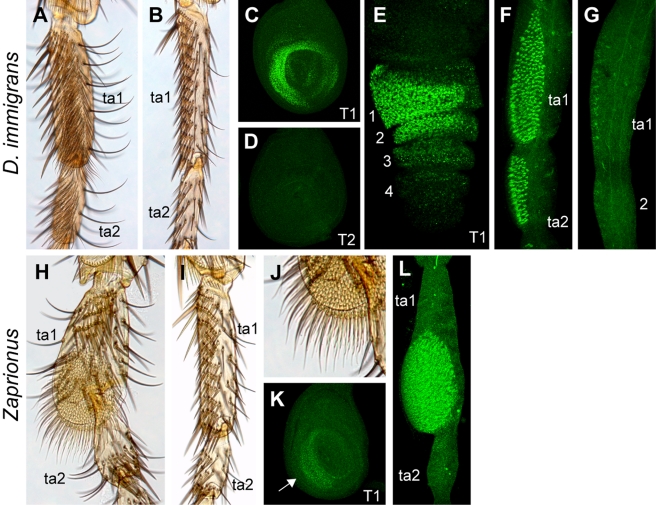
Dsx expression in species that evolved lineage-specific sexually dimorphic structures. Dsx immunostaining is in green. (A–G) *D. immigrans*. (A, B) Adult male and female T1 legs, respectively. (C) Male T1 leg disc. (D) Male T2 leg disc. (E) Male T1 prepupal leg. (F, G) Male and female T1 pupal legs, respectively, at 48 h AP. (H–L) *Zaprionus tuberculatus*. (H, I) Adult male and female T1 legs, respectively. (J) Male-specific brush structure shown at higher magnification. (K) Male T1 leg disc. Arrow, dsx expression domain. (L) Male T1 pupal leg at 48 h AP.

The sex comb is a recent evolutionary innovation that is absent in most *Drosophila* species. In the ancestral condition, the pattern of mechanosensory bristles is similar in males and females. To understand the role of *dsx* regulation in the origin of sex combs, we examined Dsx expression in several distantly related species of *Drosophila* and related genera (Figures 5, 6). The *melanogaster* and *obscura* species groups form a monophyletic lineage characterized by the presence of sex combs (Figure 5A). In *D. pseudoobscura*, a representative of the *obscura* group, Dsx is expressed in the presumptive sex comb region (Figure 5D,E), suggesting that this expression domain was already present in the last common ancestor of both species groups.

In *Scaptodrosophila lebanonensis* and *Drosophila* (*Dorsilopha*) *busckii*, which are among the most distant outgroups in our analysis, no Dsx expression is seen in the L3 leg discs (Figure 5B,C). In *D. hydei* and *D. virilis*, which represent different species groups in the subgenus *Drosophila* and also primitively lack sex combs, Dsx is expressed in the T1 tarsus in two clusters per segment during the larval and prepupal stages (Figure 5N,P and unpublished data). These clusters, which are seen in both males and females but only in the T1 leg (Figure 5O), are not homologous to the presumptive sex comb region, resembling instead the transient expression in the distal tarsal segments of male *D. melanogaster* (Figure 1F). Dsx expression in *D. hydei* and *D. virilis* is also transient: by the time of leg extension in the early pupa, when bristles begin to develop, no Dsx expression is detected in either males or females (Figure 5Q).

The closest well-studied relatives of the *melanogaster* and *obscura* species groups that lack sex combs are the Neotropical *Sophophora* including the *willistoni* and *saltans* species groups (Figure 5A) [42],[43]. However, the Neotropical *Sophophora* have recently been shown to be the sister group of the genus *Lordiphosa*, some but not all representatives of which have sex combs [8],[44]–[46]. Thus, it is not clear whether the *willistoni* and *saltans* species groups lack sex combs primitively or have lost them secondarily.

In *D. willistoni* and *D. saltans* imaginal discs, the DsxC antibody shows expression around the entire circumference of the tarsus in all three pairs of legs in both sexes, as well as in a more proximal crescent that is only seen in the male T1 disc (Figure 5G–I). Surprisingly, the ring pattern is seen with both DsxC and DsxM antibodies in both males and females, while the male T1 crescent is only detected with the DsxC antibody. Although the DsxC antibody reveals a typical *dsx* expression pattern in the adult brain of *D. willistoni* (Figure 5J), the DsxM antibody shows a different pattern, suggesting that it may not be specific to Dsx. Thus, it is not clear whether the ring seen in larval leg discs reflects *dsx* expression. At 5 h AP, this ring can be seen to extend from ta2 to ta4; the crescent pattern can no longer be detected at this stage (Figure 5K). By the time of leg extension in the early pupa (24–27 h AP), the ring pattern also disappears from the T1 legs of both sexes (Figure 5L). Thus, in contrast to the *melanogaster* and *obscura* species groups, *dsx* expression is not maintained at the developmental stage when bristle differentiation begins. The morphology and chaetotaxy of T1 and other legs in *D. willistoni* and *D. saltans* are sexually monomorphic, lacking even the male-specific chemosensory bristles that are present in most other *Drosophila* lineages (Figure 5F, not shown). This suggests that *dsx* is not directing sex-specific morphological differentiation in the legs of these species.

Overall, our results show that *dsx* is expressed in temporally dynamic, rapidly evolving, and segment-specific patterns in *Drosophila* legs. However, *dsx* expression in the presumptive sex comb region appears to be an evolutionary innovation that coincides with the origin of the sex comb.

### Independent Origin of Other Sex-Specific Structures Correlates with Gain of *dsx* Expression

Sex combs are only one example of sex-specific structures that decorate the legs of many Drosophilidae and other Diptera [47]. For example, T1 TBRs show strong sexual dimorphism in the *immigrans* species group, a member of the *Drosophila* subgenus that is distantly related to the *melanogaster* and *obscura* groups and other *Sophophora* (Figure 5A). The females of *D. immigrans* have the same arrangement of TBRs as other *Drosophila* species, while in males the anterior-ventral surface of ta1 and ta2 is covered with smaller but much more numerous and densely packed bristles (Figure 6A,B). The corresponding region of the L3 imaginal disc shows Dsx expression in both males and females (Figure 6C, not shown); in contrast, no expression is seen in T2 and T3 legs (Figure 6D). By 5 h AP, this expression remains strong in males but begins to fade in females (Figure 6E, not shown). In extended pupal legs, when bristles begin to differentiate, all of the densely packed bristles are expressing high levels of Dsx in males, whereas no expression is seen in the homologous region in females (Figure 6F,G).

In most species of the genus *Zaprionus*, TBRs on the distal ta1 of the T1 leg are replaced with much thinner and more numerous bristles that form a densely packed brush [48],[49]. This structure is only observed in males, while females retain standard T1 leg morphology (Figure 6H–J). As in the *melanogaster* and *immigrans* species groups, we find that this sex-specific pattern is prefigured by Dsx expression in the corresponding region of the T1 leg (Figure 6K,L), but no expression is seen in the T2 and T3 legs (not shown).

Phylogenetic analysis suggests that male-specific morphological structures originated independently in the *immigrans* species group, *Zaprionus*, and the *melanogaster*+*obscura* clade (Figure 5A). In each case, these morphological innovations correlate with newly evolved, T1-specific patterns of *dsx* expression. These observations suggest that the evolutionary gain of new *dsx* expression domains through a regulatory link between *Scr* and *dsx* has been a key step in the origin of novel sexually dimorphic structures.

## Discussion

### Localized Activation of *dsx* Induces the Formation of a Sex-Specific Structure

Traditional models of sexually dimorphic development in *Drosophila* have assumed that the sex determination pathway functions ubiquitously, and emphasized the joint regulation of target genes by *dsx* and the genes that establish positional information [19]. Indeed, co-regulation of downstream targets by *dsx* and spatial selector genes and signaling pathways plays a key role in the development of sex-specific morphological structures including genitalia [50]–[52], posterior abdominal segments [1],[2], and oenocytes [53]. However, recent work has shown that *dsx* is expressed in tightly restricted spatial patterns [30]–[35], suggesting that sexually dimorphic development may also be regulated through localized deployment of *dsx*. Here, we show that localized transcriptional activation of *dsx* in the T1 leg initiates the development of a sex-specific structure, and that the spatial pattern of *dsx* defines the position and morphology of this structure. For the first time, we also identify an upstream regulator of *dsx* transcription, the HOX gene *Scr*. Our results indicate that *Scr* is responsible for activating *dsx* expression in the T1 leg, and thus for restricting sexually dimorphic chaetotaxy to a single thoracic segment. Since Dsx expression is more restricted than that of Scr, we suspect that *dsx* is also regulated by one or more of the transcription factors that establish the proximo-distal leg axis.

**Figure 7:**
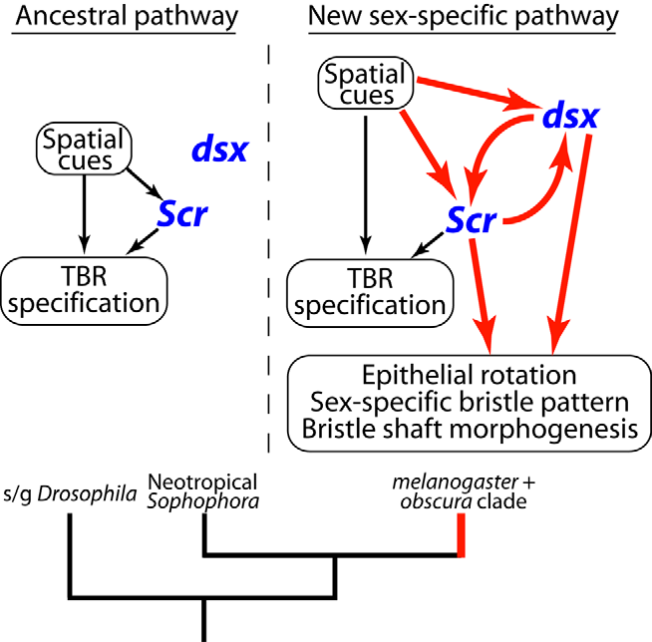
A model for the origin of a new sex-specific developmental pathway. Ancestral regulatory interactions are indicated in black, and newly evolved interactions in red. In the ancestral condition (left), leg patterning genes lay down the basic bristle pattern and establish a domain of high *Scr* expression on the ventral-anterior surface of the distal Ti and ta1. High levels of *Scr* organize the ventral-anterior bristles into TBRs. *dsx* is not expressed in the TBRs so they develop in a sexually monomorphic manner. In the *melanogaster*-*obscura* clade (right), *dsx* was recruited into the TBR development pathway under the control of both *Scr* and leg patterning genes. *Scr* activates *dsx* in T1 at the late larval stage, while *dsx* modulates *Scr* at the pupal stage to make its expression sexually dimorphic in some species. Both genes have acquired new downstream targets involved in bristle patterning and morphogenesis.

In turn, *dsx* up-regulates *Scr* in males in the presumptive sex comb region prior to and during sex comb rotation. Thus, the HOX and sex determination genes establish a positive autoregulatory loop (Figure 7). The mutual up-regulation of *Scr* and *dsxM* may explain why Dsx levels become much higher in males than in females as sex comb development progresses. The loss of Dsx expression in the homologous region in females is caused by the gradual reduction of protein levels in both epithelial and bristle cells; we do not observe large amounts of cell death in this region at the pupal stage. In contrast, Dsx-expressing domains in the central nervous system (CNS) become sexually dimorphic through programmed cell death and cell division. In one set of Dsx-expressing neurons, DsxF directs cell death in females, while in another DsxM contributes to an increase in cell division in males [33]. In the embryonic gonad, sex differences in the number of Dsx-expressing cells also result from the activation of cell death by DsxF [54]. Taken together, these results demonstrate that differences in *dsx* transcription, functional differences between Dsx isoforms, and the cellular context in which these isoforms are expressed can lead to sex-specific differentiation through a variety of cellular processes.

The molecular mechanisms responsible for the *Scr*-*dsx* feedback loop may be different at different stages. The initial activation of *dsx* by *Scr* in the late L3 leg disc may be direct, since the two proteins accumulate in the same cells. However, once the bristle precursor and epithelial cells are segregated at the pupal stage, Scr and Dsx domains become complementary and cell type-specific: Dsx expression is highest in the sex comb teeth while Scr is excluded from the bristle cells but is strongly up-regulated in the epithelial cells immediately adjacent to the sex comb. These patterns suggest that the cross-regulation between Dsx and Scr at this stage may be mediated by cell-cell signaling.

The regulation of *dsx* and *Scr* in precise spatial and cell type-specific patterns casts the roles of HOX and sex determination genes in development in a new light. Instead of modulating the output of a patterning network from the outside as “master regulators,” both *dsx* and *Scr* are intimately integrated into the middle of this network (Figure 7). Akam [55] has suggested that HOX genes may act more as “micromanagers” than master regulators in many developmental contexts. It now appears that the main determinant of sex-specific development may have to be demoted to a similar position.

### Evolutionary Origin of a Sex-Specific Developmental Pathway

New sex-specific traits may arise in two different ways. If the sex determination pathway is already active in the relevant tissue, the origin of a novel trait requires only the acquisition of new joint downstream targets by the sex determination and spatial patterning genes. This may happen either through evolution of Dsx binding sites in a previously sexually monomorphic enhancer or through the co-option of a pre-existing dimorphic enhancer into a new tissue [2],[53]. In contrast, a tissue that shows no sexual dimorphism in the ancestral condition may not express *dsx* at all. In this case, a new sex-specific trait cannot arise without the evolution of a new *dsx* expression domain. To our knowledge, the sex comb is the first example of an evolutionary change of this kind. We suggest that in the common ancestor of the *melanogaster* and *obscura* species groups, *dsx* was recruited into a previously sexually monomorphic developmental pathway, resulting in the gain of a novel expression domain in the presumptive sex comb region (Figure 7). This cooption may have been facilitated by the fact that *dsx* is already expressed in segment-specific, and presumably *Scr*-regulated patterns in some species that primitively lack sex combs. In parallel, *Scr* and *dsx* must have acquired new joint downstream targets that mediate different aspects of sex comb morphogenesis including bristle patterning, tissue rotation, and modification of bristle shafts. Subsequent changes in the spatial regulation and cross-regulation of *dsx* and *Scr*, as well as gains and losses of downstream targets, have likely contributed to the dramatic evolutionary diversification of sex combs.

### Positive Feedback and Evolvability

The positive feedback loop between *dsx* and *Scr* may play a major role in generating sex comb diversity across species. Regardless of the exact molecular mechanism, our results suggest that any alteration in *Scr* expression expands or contracts the *dsx* domain, and vice versa. One can imagine that any mutation that increases *Scr* expression, for example a *cis*-regulatory mutation in the *Scr* leg enhancer, would increase the expression of *dsx*, which in turn would further up-regulate *Scr* in the male, and so on; the effects of any mutation that increases the expression of *dsx* would be similarly amplified. Conversely, mutations that reduce either *Scr* or *dsx* expression would also have their effects on both *Scr* and *dsx* magnified by the autoregulatory loop. This positive-feedback amplification would allow sex comb morphology to respond rapidly to selection for increased or decreased sex comb size. Comparative and experimental analyses show that male secondary sexual traits are lost (or reduced) as frequently as acquired (or exaggerated), and that this pattern may be due to rapidly shifting female preferences [56],[57]. It is possible that positive feedback loops similar to the *Scr*-*dsx* circuit are involved in the rapid gain, diversification, and loss of other exaggerated display characters and sexually selected traits.

### A General Role for *dsx* in Evolutionary Innovations?

The spatial regulation of *dsx* in *Drosophila* raises an intriguing question about the evolution of sex-specific traits in general. Sexual selection leads not only to the rapid evolution of existing characters, but also to the frequent origin of novel morphological structures, behaviors, and other phenotypes [58],[59]. Almost every lineage of animals has invented its own sex-specific (often, but not always, male-limited) organs. In Diptera, different families and genera have evolved a variety of sex-specific structures and modifications on all three pairs of legs, on the eyes, mouthparts, and the head capsule, on the thorax, abdomen, and generally on every body part imaginable [47],[60],[61]. Some of these structures reach truly bizarre appearance and proportions, such as the branched and malformed legs of some Dolichopodidae and Platypezidae or the eye stalks that exceed body length in Diopsidae and Platystomatidae, yet they have no clear homologues outside of the lineages that possess them. At the same time, the loss of sex-specific characters occurs at roughly the same rate as the origin of new ones [56]—in other words, there is a constant turnover of sex-specific traits.

Is it possible that the proximate cause of this turnover of sex-specific traits lies in the acquisition and loss of new spatial expression domains of *dsx*? This model is supported by our observation that different male-specific structures that independently evolved in the *immigrans* species group and in the genus *Zaprionus* are, like the sex comb, associated with the origin of new *dsx* expression patterns. Male-specific reduction of wing size in *Nasonia* wasps, which is associated with genetic changes near the *dsx* locus, may represent another example [62]. The modular organization of transcriptional control allows gene expression in different tissues to be decoupled both functionally and evolutionarily through the use of modular, tissue-specific enhancers [63],[64], making the gain and loss of discrete expression domains entirely possible. A *dsx* enhancer responsible for sex comb development in *Drosophila* was gained and underwent rapid diversification within the genus, raising the possibility that other novel enhancers and expression domains have originated in other lineages on similarly short timescales.

## Materials and Methods

### Drosophila Strains

The following strains were used: *rn-Gal4^4–5^* [65], *neur-Gal4^A101^* [66], *UAS-dsxM* (Lee et al. 2002) [30], *UAS-Scr^M15^* [67], *UAS-dsxRNAi*, *UAS-ScrRNAi* [68], and *tub*-*Gal80^ts20^* [69]. Expression of the UAS constructs was activated at the wandering third instar or white prepupal stage by shifting *tub*-*Gal80^ts20^*; Gal4/UAS flies from 18°C to 30°C.

### Immunocytochemistry and Imaging

Animals were reared, processed for immunocytochemistry, and imaged as described [16],[33]. The primary antibodies used were rat anti-DsxCommon, 1∶50 [33], rat anti-DsxM, 1∶500 [31], and mouse anti-Scr 6H4.1, 1∶10 [39]. The secondary antibodies were AlexaFluor 488 and 594 used at 1∶200 (Invitrogen, Carlsbad, CA). In *D. melanogaster*, both Dsx antibodies showed identical expression patterns in larval leg discs and pupal legs. In species distantly related to *D. melanogaster*, cross-reactivity of the Dsx antibodies was confirmed by staining adult male and female brains. The DsxC antibody identified neuronal clusters that were similar in size and position to those seen in *D. melanogaster* (Figure 5), while the DsxM antibody showed variable staining in different species suggesting that it may not be fully specific. With the exception of *D. melanogaster*, all Dsx expression patterns shown in the figures were determined using the DsxC antibody.

### In Situ Hybridization

In situ hybridization on pupal legs and imaginal discs was performed as described [37] using RNA probes directed against the male-specific exon of *dsx*. Probe template was amplified from genomic DNA by PCR using primers dsxM-Fwd (AATCGCACTGTAGCCCAGATC) and dsxM-Rev (CTGGAGTCGGTGGACAAATC).

## Abbreviations

CNS: central nervous system
*dsx*: *doublesex*
DsxC: common domain of Dsx
*dsxF*: doublesex female isoform
*dsxM*: doublesex male isoform
*Scr*: *Sex combs reduced*
TBRs: transverse bristle rows
